# Gene Coexpression and Evolutionary Conservation Analysis of the Human Preimplantation Embryos

**DOI:** 10.1155/2015/316735

**Published:** 2015-07-27

**Authors:** Tiancheng Liu, Lin Yu, Guohui Ding, Zhen Wang, Lei Liu, Hong Li, Yixue Li

**Affiliations:** ^1^Key Laboratory of Systems Biology, Shanghai Institutes for Biological Sciences, Chinese Academy of Sciences, 320 Yueyang Road, Shanghai 200031, China; ^2^Key Laboratory of Contraceptive Drugs and Devices of National Population and Family Planning Commission of China, Shanghai Institute of Planned Parenthood Research, 2140 Xietu Road, Shanghai 200032, China; ^3^Shanghai Center for Bioinformation Technology, 1278 Keyuan Road, Shanghai 201203, China

## Abstract

Evolutionary developmental biology (EVO-DEVO) tries to decode evolutionary constraints on the stages of embryonic development. Two models—the “funnel-like” model and the “hourglass” model—have been proposed by investigators to illustrate the fluctuation of selective pressure on these stages. However, selective indices of stages corresponding to mammalian preimplantation embryonic development (PED) were undetected in previous studies. Based on single cell RNA sequencing of stages during human PED, we used coexpression method to identify gene modules activated in each of these stages. Through measuring the evolutionary indices of gene modules belonging to each stage, we observed change pattern of selective constraints on PED for the first time. The selective pressure decreases from the zygote stage to the 4-cell stage and increases at the 8-cell stage and then decreases again from 8-cell stage to the late blastocyst stages. Previous EVO-DEVO studies concerning the whole embryo development neglected the fluctuation of selective pressure in these earlier stages, and the fluctuation was potentially correlated with events of earlier stages, such as zygote genome activation (ZGA). Such oscillation in an earlier stage would further affect models of the evolutionary constraints on whole embryo development. Therefore, these earlier stages should be measured intensively in future EVO-DEVO studies.

## 1. Introduction

Evolutionary developmental biology (EVO-DEVO) studies how the dynamics of development affect the phenotypic variation arising from genetic variation and its correlation with phenotypic evolution. In this subject there is a central issue: which is the most conserved period or the crucial section during the entire developmental process of an organism. While it is unarguable that the later stages of embryogenesis are not conserved among species, two major models have been proposed: the “funnel-like” model, in which the earliest embryo shows the most conserved pattern, and the “hourglass” model, in which the middle point of development is imposed with the most evolutionary constraints [1]. The “hourglass” model, which assumes the midembryonic stage (phylotypic), which shows developmental constraints and functional importance, was originally proposed due to the expression of Hox genes in middle point of vertebrate development [2] and has been preferred in comparative transcriptomic studies nowadays [3, 4]. In addition to the transcriptomic similarity of phylotypic stages between different species, transcriptome age index (TAI) based methods, which address the total evolutionary ages of expressed genes in each developmental stage, show convergent evolution matching an hourglass pattern of embryogenesis in animals and plants [5, 6].

Mammalian preimplantation embryonic development (PED) starts from fertilization and ends at implantation of the embryo in the endometrial lining of the uterus [7]. After fertilization, the major genetic substances in the transcriptome of the zygote are the maternally deposited transcripts. After 2-3 rounds of cell divisions, maternally inherited transcripts are degraded gradually and new transcripts of zygote are produced by the new diploid nucleus. This process is termed zygote genome activation (ZGA) [8]. These changes are not easily captured by traditional gene expression microarray techniques, as the sensitivity of microarray technology is limited when detecting low expressed genes or expression in a single cell [5, 9]. With the development of single cell RNA sequencing technology [10], we were able to identify precisely gene expression changes during the embryo developmental process which are unapparent in the microarray analysis [5]. In order to illustrate the earliest developmental gene expression fluctuation of PED which contain the crucial ZGA process and may further affect the later developmental stages, it is meaningful to look into these PED stages and identify the genetic modules regulating in each period of PED [11].

From an EVO-DEVO viewpoint, inspection of the selective constrains in PED is interesting because the trend in this period would further influence the tendency of evolutionary constrains in the middle stages of embryo development such as the phylotypic stage. Despite the importance of the expression profile of the PED stage, previous comparative transcriptome research has yet to characterize it [3, 5]. The lack of understanding of these PED stages has led past researchers to conclude that selective constraints during the earlier developmental stages increase continuously in the “funnel-like” model while they decrease in the “hourglass” model. Analysis of the selective constrains of genes in each PED stage could aid in distinguishing between the formation mechanism of the “hourglass” model or the “funnel-like” model and also consummate the whole pattern of selective constrains that act on embryonic development.

Based on the single cell RNA sequencing results of human preimplantation embryos from the oocyte stage to late blastocyst stage [12]. Applying weighted gene coexpression network analysis (WGCNA) [13], we were able to identify representative genes in each stage and summarized selective pressure on these genes to clarify the selective trend in earlier developmental stages. We found certain patterns of the evolutionary constraints that imposed on different stages of human preimplantation embryos; therefore we illustrated selective constraints on PED stages, which also presented fluctuation properties, considering that these earlier stages should be included for studying the constraints on the whole embryo development.

## 2. Results

### 2.1. Coexpression Modules for Stages in Human Preimplantation Embryos

In the course of evolution, most biodiversity is due to alterations in gene regulation relationships rather than the sequence mutations on genes [14]. Coexpression gene modules tend to evolve together so as to share evolutionary patterns [15]. Therefore, we used gene coexpression analysis rather than differential expression to identify genes that may have close regulation relationships [16].

**Figure 1:**
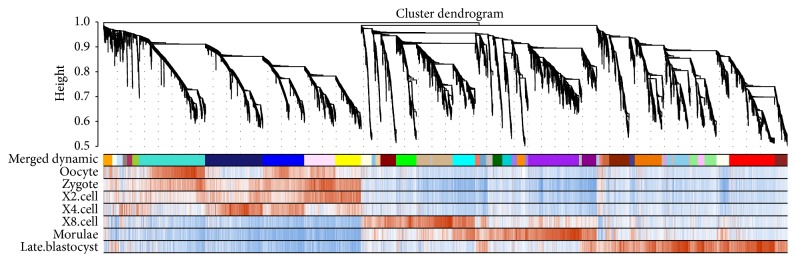
Coexpression gene modules in human preimplantation embryonic development. Hierarchical cluster tree shows the coexpression modules identified by WGCNA. The panels from top to bottom are merged dynamic modules labeled with different colors and genes correlation with indicators of each stage. The red means positive correlation while blue means negative correlation, and correlation coefficients are in direct proportion to the color depth.

In order to study selective constrains in preimplantation embryonic development, we analyzed the transcriptome profiles of human preimplantation embryos (including oocyte, zygote, 2-cell, 4-cell, 8-cell, morulae, and late blastocyst stages) that were obtained by single cell RNA sequencing. Stage-specific coexpression modules were selected by the gene coexpression network analysis (WGCNA) (Figure 1) which is an unsupervised clustering method to group genes which have coexpression patterns into distinct modules [13]. This is a reliable gene coexpression analysis tool and is wildly adopted by many investigators [17–20]. After merging correlated modules with a stringent threshold, we assigned 27 out of 41 modules into a specific preimplantation developmental stage according to the correlation of eigengene of every module with each stage indicator (*r* > 0.6, *P* < 0.001). Some modules might correlate with two adjacent developmental stages because of the similarity of these two adjacent developmental stages. To remove the bias of stage comparison, we assigned this kind of modules to stage in which they had the highest correlation coefficient. After that, each module was classified into a specific developmental stage and most of the genes in each module showed consistent overexpressed behavior in corresponding developmental stage (Figure 1).

**Figure 2:**
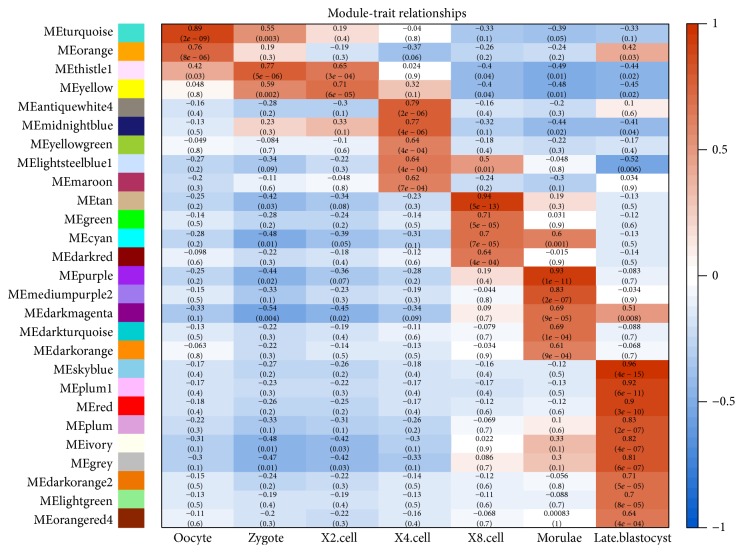
Modules are distributed to each stage according to the correlation between eigengenes and stage indicators. The red means positive correlation while blue means negative correlation, and correlation coefficients are in direct proportion to the color depth as shown in right side. Each element of the matrix denotes the correlation coefficients between module eigengenes (row) and stage (column); then the significant level of correlation is marked below the correlation coefficients.

Genes in multiple modules of the same stage were merged together. In total, we obtained 2 coexpression modules for the oocyte stage, 1 module for the zygote stage, 1 module for the 2-cell stage, 5 modules for the 4-cell stage, 4 modules for the 8-cell stage, 5 modules for the morulae stage, and 9 modules for the late blastocyst stage (Figure 2). We obtained 1409, 583, 481, 1494, 1731, 1720, and 3132 specific genes for each stage, respectively. The large number of genes in the oocyte showed a complicated regulation mechanism that involved the expression of maternal genes. The number of coexpression modules and genes gradually increased with the progress of zygote development, which implied the formation of embryo complexity and modularity.

### 2.2. Validation of the Biological Function for Modules

We further investigated the biological functions of genes in each specific stage by using DAVID software [21]. Gene ontology biological process (GOBP) enrichment analysis showed that genes from each stage were enriched in the relevant functions of corresponding developmental process. We also verified the function of genes in each stage by comparing them with the known function categories that were identified by Xue et al. on a different dataset of human preimplantation embryos [11]. And we compared them with the functional term identified by different methods on same datasets [12] (Table 1). The zygote gene activation (ZGA) process, which is the principal transformation of the pre-implantation period, was endorsed by significant overrepresentation of genes involved in transcription and transcription regulation process from 4-cell stage to morulae stage. In the late blastocyst stage, genes were significantly enriched in protein translation and function-associated pathways such as protein localization, transport, and phosphorylation.

### 2.3. Various Selective Pressures on Gene Sequence

**Figure 3:**
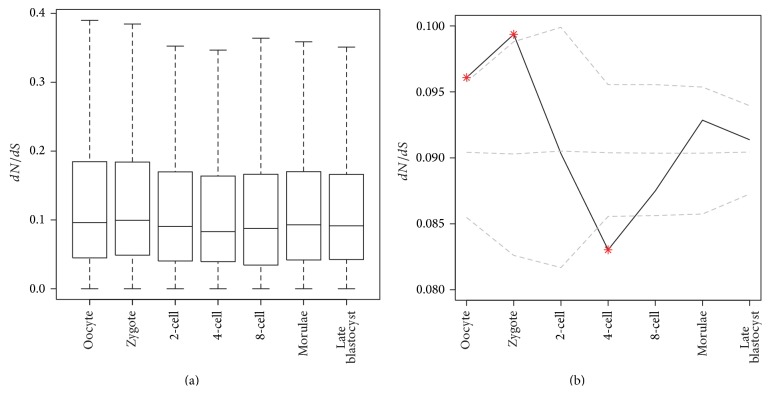
(a) Box plot of conserved index during different development stages. Conservation is measured by the nonsynonymous to synonymous substitution ratios (*dN*/*dS*). (b) The median of *dN*/*dS* ratio in each stage is represented by the solid line. The dash lines denote the median and the 95% confidence interval for the *dN*/*dS* ratio of randomly selected genes, which have same number of genes in corresponding stage. The asterisks denote that *dN*/*dS* are significantly different (*P* < 0.05) from the median of background.

The nonsynonymous to synonymous substitution ratio (*dN*/*dS*) is a widely used method to measure gene sequence conservation [22]. We used *dN*/*dS* ratio for genes in each module to quantify the selective pressure on the corresponding developmental stage. The *dN*/*dS* ratios were calculated between mouse and human, as we intended to measure the pressure acting on sequence of genes in the mammalian species. Then their distributions were illustrated in Figure 3(a). Next we randomly sampled same number genes within each stage and calculated the median of the *dN*/*dS* distribution for the random dataset, which stood for the background.  Figure 3(b) shows the median of *dN*/*dS* for genes in coexpression modules and the median of *dN*/*dS* for randomly selected genes.

The *dN*/*dS* ratios of stage-specific genes gradually decreased until the 4-cell stage, which may be caused by the consumption of the maternal genes and the expression of new genes of the zygote itself as shown by previous studies of preimplantation embryos [11, 12, 23–26]. Oocytes and zygotes had a higher median of *dN*/*dS* ratios relative to the median *dN*/*dS* for all genes whereas the *dN*/*dS* ratios of genes belonging to the 4-cell stage were significantly lower than the median *dN*/*dS* for all genes. From the zygote stage to the turning point 4-cell stage, the decreasing trend of *dN*/*dS* ratios was parallel with the process that the maternal genes expended and zygote genes emerged. The genes of the 4-cell stage were more inclined to be expressed by zygotes and had low *dN*/*dS* ratios. At the same time, genes regulated in the zygote or oocyte stages were left by maternal source and these genes had high *dN*/*dS* ratios. So the decreasing trend from maternity to zygote might suggest more striking selective pressure acting on the genes produced by zygote than selective pressure effecting genes inherited from maternity [27]. After the 4-cell stage we detected a pattern of increasing *dN*/*dS* ratios, which shows these stages expressed genes with selective pressure not as strong as 4-cell stage.

### 2.4. Stage-Specific Genes Were Born in Different Ancient Roots

**Figure 4:**
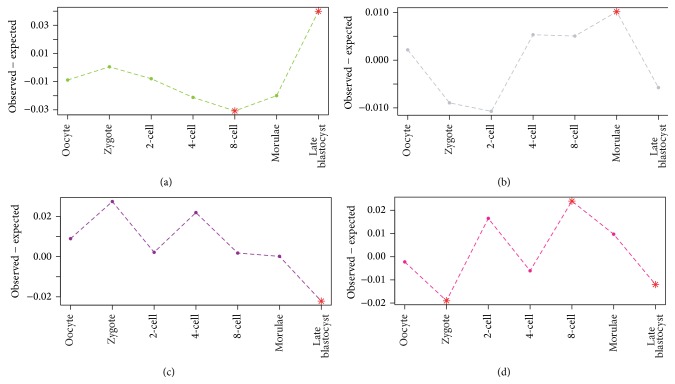
Distributions of gene ages. Genes were classified into four groups based on their first appearance in the phylogeny: (a) Opisthokonta-Bilateria, (b) Sarcopterygii-Amniota, (c) Chordata-Euteleostomi, and (d) Mammalia-Eutheria. For each stage, the vertical axis shows the observed frequencies minus expected frequencies of gene ages. The asterisks denote significant enrichment (*P* < 0.01) in a specific gene group for each stage.

The ages of stage-specific genes have been used as indices of evolutionary constraint [5]. We traced the root of every gene expressed in human preimplantation embryo in the phylogeny and used the ancient level of the root to represent the conservation of the gene. Based on the phylogenetic taxonomy of their roots, genes were separated into four groups: (1) Opisthokonta-Bilateria, (2) Sarcopterygii-Amniota, (3) Chordata-Euteleostomi, and (4) Mammalia-Eutheria. For each gene set, the number of genes in each of the 4 groups was calculated to represent the age distribution. Next we marked every preimplantation developmental stage with a specific age distribution and used the age distribution of all genes as background. To detect the difference of gene age during different development stages, the age distribution of genes in each stage was compared with the background distribution of all genes (Figure 4).

We detected a clear changing trend for the Opisthokonta-Bilateria genes, with their proportion decreasing from the zygote to the 8-cell stage and then increasing until the late blastocyst. In particular, the genes belonging to the 8-cell stage were significantly depleted in Opisthokonta-Bilateria and overrepresented in Mammalia-Eutheria, which implied most genes expressed in this stage are recently born in the Mammalia-Eutheria lineage compared to other stages. In other words, these new genes, which were expressed and regulated as modules in 8-cell stage, were products of developmental evolution in the Mammalia-Eutheria lineage. This suggested that genes expressed in the 8-cell stage had a crucial function for the ZGA process of organism in Mammalia-Eutheria lineage [11, 28, 29].

As Figure 3 shows, after the 8-cell stage there was an opposite trend of increasing Opisthokonta-Bilateria genes from depletion to overrepresentation and decreasing Mammalia-Eutheria genes from overrepresentation to depletion. Finally the late blastocyst stage showed the opposite pattern—the stage was significantly depleted genes belonging to Mammalia-Eutheria and Chordata-Euteleostomi groups and it was overrepresented of genes in Opisthokonta-Bilateria group. This sort of opposite pattern illustrated that the late blastocyst stage was conserved as it tended to express the oldest genes.

### 2.5. Genes in Each Stages Present Diverse Duplicated States

**Figure 5:**
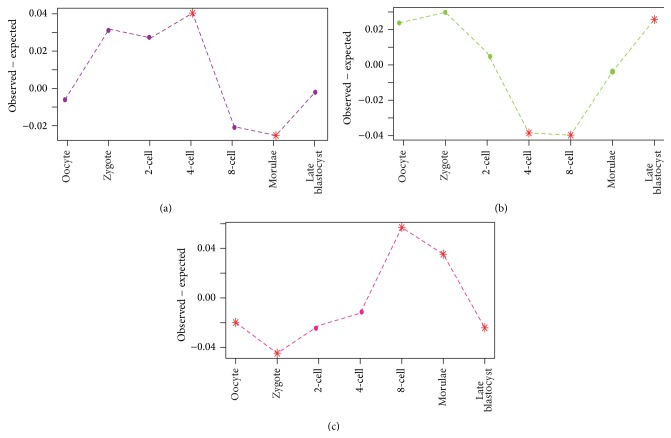
Distributions of different gene ortholog types comparing human with zebra fish: (a) one-to-many ortholog, (b) one-to-one ortholog, and (c) new genes. For each stage, the vertical axis shows the observed proportions minus expected proportions of genes in each ortholog type. The asterisks denote significant enrichment (*P* < 0.01) in a specific ortholog scenario for each stage.

Gene duplication state is also an indicator of selective pressure [30]. In order to evaluate the conservation of genes more widely, we chose the zebra fish, an evolutionary distant species, as reference to check the gene-duplicated situation of human genes in each development stage. Genes were separated into four groups based on the gene duplication states: (1) one-to-many, (2) one-to-one, (3) many-to-many, and (4) new gene (no ortholog in the zebra fish genome). We removed the many-to-many gene pairs because it is difficult to evaluate their conservation. As stated above, we compared the observed distribution of genes in each stage with the expected distribution that was recorded by distributing all genes into these 3 groups (Figure 5).

Genes falling in one-to-many orthologs revealed that they were single copy in human and their orthologs had duplications in zebra fish. Knowing that constrained developmental stages should display less change in gene family size [31], the genes, which duplicate in other species but keep singleton in human developmental stages, should be considered to be conserved specifically in* Homo sapiens*. Otherwise, the one-to-one orthologs retain the functions of ancestral gene since the last shared common ancestor, and left no duplication in the human or zebra fish lineage. Therefore genes of one-to-one orthologs also should be subject to functional constraints. Just as in the above age analysis of genes, the new genes, which were new products during the evolutionary process of* Homo sapiens*, were considered to be under less constraint. At last, many-to-many orthologs showed duplication events in both species and their conservation patterns were complicated; thus we ignored many-to-many orthologs in the further analysis.

As Figure 5(b) shows, the one-to-one (single-copy) orthologs and new genes exhibited opposite trends in the preimplantation period which implied the transformation of evolutionary constraints on different stages during the developmental process. In particular, the 4-cell stage showed significant depletion of the one-to-one genes but overrepresentation of the one-to-many genes that signify genes of this stage is under strong functional constraints on their sequence in* Homo sapiens*. It accorded with the *dN*/*dS* result showing that genes expressed in the 4-cell stage had significantly lower human-mouse *dN*/*dS* ratios and lends further evidence to the hypothesis of conservation of genes belonging to the 4-cell stage. Moreover, the 8-cell stage showed overrepresentation of the newborn genes and depletion of the one-to-one genes, which is consistent with the gene age analysis. The large number of new genes in the 8-cell stage offers further evidence for human-specific embryonic development occurring in this stage [11, 28, 29].

As with the above gene age analysis, we also detected conserved convergence from the 8-cell stage to late blastocyst stage reflected by the transition from an overrepresented state to a depleted state of the new genes and by the transition from depleted state to overrepresented state of the one-to-one genes. Finally, the late blastocyst stage reached a conserved state, which was significantly depleted of new genes and overrepresented for one-to-one genes.

### 2.6. Evolvability of Regulatory Regions in Upstream of Stage-Specific Genes

**Figure 6:**
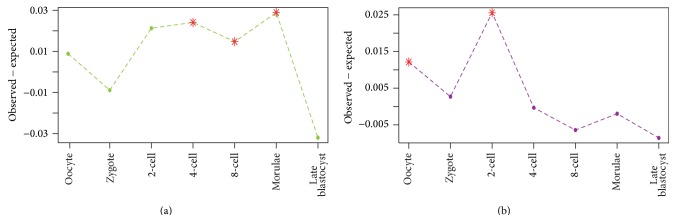
(a) Distributions of transcription factors (TFs) in stage-specific modules. (b) Distributions of genes with highly conserved noncoding elements (HCNEs) in their* cis*-regulatory regions. For each stage, the vertical axis shows the observed proportions minus expected proportions of TF and genes with HCNEs, respectively. The asterisks denote significant enrichment (*P* < 0.01).

Conservation of* cis*-regulatory sequences is also a critical standard for measuring the selective pressure on genes [14, 32], and highly conserved noncoding elements (HCNEs) are often considered to be associated with developmental regulatory genes or transcription factors (TFs) [23, 33]. Therefore, we determined the transcriptional importance of stage-specific genes by analyzing their potential to become TFs and the distribution of HCNEs in their promoter regions (Figure 6).

We found that promoter regions of genes in the 2-cell stage were significantly enriched for HCNEs, and there are more TFs in 2-cell stage than expected. These enriched transcriptional factors and transcriptional regulatory elements may promote effective gene transcripts in the 2-cell embryo and launch the progress of zygote genome activation (ZGA). TFs were significantly enriched in 4-cell, 8-cell, and morulae stages, which indicated that the gene expression and regulation network became more sophisticated during the zygote gene activation (ZGA) process. Our finding of a relatively desolate transcriptional scenario in the late blastocyst stage accords with the findings of Piasecka et al. [31], who proposed that the cleavage/blastula modules of zebra fish development are not enriched with transcriptional devices. Finally, the gathering of these transcriptional elements during the ZGA process could not be disregarded and this might further influence the evolutionary model or regulation mechanism of the whole developmental schedule.

### 2.7. Patterns of Evolutionary Constraints in Preimplantation Embryonic Development

Based on WGCNA, we clustered the genes of human preimplantation embryonic development into modules and linked these modules to specific stages of this developmental process. Next, we checked four conservation properties for stage-specific genes, including gene sequences, gene ages, gene orthologs, and regulatory elements. All of these indices implied several features during the process of human preimplantation embryonic development.

First, we observed that maternal genes were under less selective constraints while there were strong selective constraints effecting on earlier zygote-activated genes. This was verified via the reduction of the *dN*/*dS* ratio accompanied by the consumption of maternal mRNAs and ZAG expressing from zygote to 4-cell stage (Figure 3), and the over-representation of the conserved one-to-many orthologs in the 4-cell stage (Figure 5). Secondly, we discovered a switch of the evolutionary constraints at 8-cell stage in which the embryo tended to express new genes. This trend is reflected by the fact that *dN*/*dS* begin elevating after the 8-cell stage (Figure 3). Meanwhile, genes in 8-cell stage present depletion of oldest Opisthokonta-Bilateria genes and show overrepresented of newest Mammalia-Eutheria genes (Figure 4). The burst of new genes in the 8-cell stage was further demonstrated by the depletion of one-to-one zebra fish orthologs genes and overrepresentation of human specific genes in this stage (Figure 5). Lastly, the selective pressure on late blastocyst stage tended to increase again. The late blastocyst stage was overrepresented in the oldest Opisthokonta-Bilateria genes (Figure 4) and one-to-one orthologs of zebra fish (Figure 5). The phylotypic stage in middle development was specifically enriched for transcriptional elements so that the transcriptional factory was subtly working in this stage [31]. Our work revealed that ZGA in early stages also showed the enrichment of transcriptional elements (Figure 6), which indicated the ZGA process is under precise regulation as phylotypic stage.

**Figure 7:**
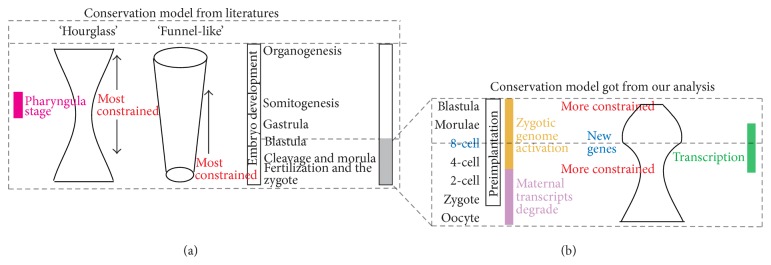
(a) Two models—the “funnel-like” model and the “hourglass” model, revealed by the evolutionary constraint on the whole developmental process of organism. Corresponding stages are labeled on the right side and earlier stages to late stages are in bottom to up sequence. Pharyngula stage (phylotypic stage) is displayed by pink rectangle on the left side. (b) The fluctuation of evolutionary constraints on the stages of human preimplantation embryonic development. In the left side, preimplantation stages are ranked from bottom to up. Maternal transcripts degrade and ZGA are labeled with purple rectangle and yellow rectangle, respectively.

In summary, we found that, in the earlier developmental stages of the human embryo, the conservation indices presented the sequence of increasing—decreasing—increasing (Figure 7), rather than increasing or decreasing monotonically. And this trend is potentially correlated with the maternal transcripts degrade and ZGA. As part of the developmental process, earlier embryo development turns out to be a complicated process which also involves the fluctuation of selective pressure.

## 3. Discussion

Mammalian developments comprise three important processes: zygote genome activation (ZGA) at earlier stages [11], expression of Hox genes at middle stages [2], and morphological formation at late stages [34]. The evolutionary conservation of these three stages has been debated at length. Previous EVO-DEVO studies concerning development [1, 4, 5, 31] focused on conservation during the whole development process, while changes of selective pressure during earlier development were neglected. These studies typically used one stage (such as the zygote stage) to represent the earlier embryonic stages [5]. Most stages of the earlier embryo (such as 2-cell to 8-cell stages) were discarded as they are relatively short compared to the long time interval of stages in middle and late embryo. By monitoring these transient stages of earlier development, the exquisite regulation mechanism of the ZGA process could be revealed [11, 12]. Rather than monitoring once after certain time intervals [4], the detecting time points should be chosen according to developmental events and time interval of different stages should be specially selected. Therefore it is meaningful to set more observing points during the earlier stages [35]. Here we analyze eight stages in preimplantation embryonic development. Our results show that the conservation scenario of the earlier embryo has a degree of fluctuation different from the direct increase or decrease previously reported [1, 3, 5, 6, 31]. The fluctuation in PED stages was probably associated with the events occurring during these stages. For instance, more selective pressure was on early zygotic genes than maternal genes [27]; therefore the measure of conservation increased during the maternal transcripts degrading process. Then the embryo was building the infrastructure so that it expressed highly conserved genes during the 2-cell stage to 4-cell stage. After this, the embryo expressed some species specific genes to determine its fate inclination [36]. Finally, the genes expressed near middle embryonic stages presented to be conserved, which was accordance with the hourglass model. Our work illustrates that the dynamics of evolutionary indices during these short-time early stages should also be taken into consideration in discussions of the “hourglass” model or the “funnel-like” model of embryo development. We believe a precise exploration of the evolutionary indices of earlier developmental stages will lead to the creation of a more sophisticated model of selective pressure on the whole development process.

## 4. Methods

### 4.1. Transcriptional Profiling of Preimplantation Embryos

The gene expression profilings of human preimplantation embryos were downloaded from NCBI's Gene Expression Omnibus [37] (http://www.ncbi.nlm.nih.gov/geo/query/acc.cgi?acc=GSE36552). It contained the whole transcriptomic RNA expression levels of oocyte stage, zygote stage, 2-cell stage, 4-cell stage, 8-cell stage, morulae stage, and late blastocyst stage, which were measured by RPKM (Reads Per Kilobase of transcript per Million mapped reads) via single cell RNA sequencing [38]. Each stage was composed of 4 biologically replicated samples except for oocyte and zygote stages, which had 3 samples. To eliminate the bias of genes which have zero or extremely low expression levels in many stages, genes with low expression in all stages (average RPKM < 0.5) were removed. Each gene symbol of the whole profile was mapped to its corresponding Ensembl gene ID and the gene symbols that have no corresponding Ensembl gene ID were discarded to reduce the potential noise. At last the expression profiles in each sample were processed by quantile normalization that accounts for different amounts of RNA present throughout embryo earlier development.

### 4.2. Weighted Gene Coexpression Network Analysis

The final expression matrix was proceed by the step by step WGCNA [13] method. First, we built a matrix which includes pairwise correlation coefficients between all pairs of genes [39]. Next, with the power of 12 which is the default value, the adjacency matrix was constructed. Depending on the resulting adjacency matrix, we calculated the topological overlap matrix, which measures the interconnectedness of the coexpression network [40]. And then this topological overlap matrix was used to perform hierarchical clustering in which genes with coexpression relationships were grouped together and formed a gene clustering tree. The primary modules were identified by Dynamic Hybrid Tree Cut algorithm [41] to cut the hierarchal clustering tree with coefficients deepSplit = 4. At last, we calculated the correlation coefficients of each pair of module eigengenes that stand for the first principal component of the module and merged highly similar modules by a stringent threshold (correlation > 0.9).

After identifying the coexpression modules, we associated these modules with specific embryo developmental stage and picked hub genes for each module. This process was based on correlating each module eigengene which represented each module with the stage indicator genes and all genes on the matrix. For genes with high correlation coefficients (correlation above 0.9 and *P* value < 0.01) with specific module, we treated them as the hub genes of this module. To associate these modules with developmental stage, we used a threshold (correlation coefficient > 0.7 and *P* value < 0.01) to pick up modules which belong to a certain stage. Modules that correlated with two stages were only kept in the stage with the highest correlation coefficients.

### 4.3. Gene Ontology Analysis

Functional annotation was performed with the DAVID Bioinformatics Resources. To correct multiple testing, the Bonferroni correction was applied. And the enriched GO biological process categories were picked up by the corrected *P* value (<0.01). Then we checked whether these enriched GO categories were also presented in the same stages of similar studies [11, 12].

### 4.4. *dN*/*dS* Analysis

We downloaded *dN* and *dS* values of all human genes using BioMart [42], which was calculated by the ortholog genes between human and mouse. After removing genes that were not presented on the expression matrix, we got 12865 *dN*/*dS* value.

We calculated the median *dN*/*dS* ratio of genes in each stage and evaluated if it was significantly higher (lower) than randomly selected genes. For each stage which has k genes, we generated 10000 sets of k randomly chosen genes from the background 12865 genes and calculated the median *dN*/*dS* ratio for each random set. *P* value was calculated as the tail probability of real *dN*/*dS* ratio in the distribution of randomly generated *dN*/*dS*.

### 4.5. Gene Age Analysis

Genes of* Homo sapiens* originated in different taxonomic root of the phylogeny so that genes have different age index. We could label every gene with an age index by its first appearance in the phylogeny. For each gene in our expression matrix the oldest node of its gene tree was retrieved from Ensembl release 75 [43] by Ensembl comparative genomics API. After that, each gene was marked with a unique age index from oldest Opisthokonta node to the latest human node.

In order to make subsequent test convincible we removed some genes falling in age interval from Eutheria to human because the number of genes in these interval is very rare (less than 5) which will obscure the statistical test. And the rest of the genes were merged into one of the following age intervals: Opisthokonta-Bilateria, Opisthokonta-Bilateria, Sarcopterygii-Amniota, and Mammalia-Eutheria. That made each category have sufficient number of genes to perform the statistical test.

Next, for every module we collected all the age indices of its k genes and counted the number of genes falling to each age interval. Then the age index distribution of expected background was estimated by classifying all genes (11919 genes) presented on the expression matrix into these categories. The number of genes in each age category was transformed to the proportion by dividing the number of all expressed genes in this stage. Then we plotted the “observed minus expected” proportions of each stage and performed Fisher's exact test to compare the observed and expected numbers of age indices in each stage. We picked up stages in which genes with certain age index were overrepresented or underrepresented (*P* value < 0.01) and highlighted these stages on the plot.

### 4.6. Human-Zebra Fish Orthologous Genes

Based on an evolutional distant species zebra fish, homology information between human and zebra fish genes was retrieved from Ensembl release 75 [43] by BioMart [42]. 10919 of the 12865 genes presented in the expression matrix have human-zebra fish paired orthologs, including 7265 one-to-one orthologs, 2995 one-to-many orthologs, and 659 many-to-many orthologs. Then the remaining 1946 human genes which do not have ortholog relationship with zebra fish genome were labeled as new gene in human.

We calculated the observed number of stage-specific genes that were in the three kinds of ortholog types (one-to-one, one-to-many, and no orthology), and constructed the expected background distribution from all genes. For each category we plotted the “observed minus expected” proportions of each stage and performed Fisher's exact test to compare the observed and expected numbers.

### 4.7. Gene Transcriptional Region Analysis

Gene transcription analysis was based on the number of transcription factors (TFs) and highly conserved noncoding elements (HCNEs) in the promoter regions of genes. Genes with GO category annotation (GO: 0006355, regulation of transcription, DNA-dependent) were defined as TFs. Location data of HCNE between human and mouse with identity above 90% was downloaded from Ancora (http://ancora.genereg.net/downloads/hg19/vs_mouse/) [33]. For each of the 12865 genes considered in our analysis, we checked if there were HCNEs located in 500 base-pairs upstream from the transcription start site. Totally we annotated 1438 and 848 genes as TFs and HCNEs, respectively. For every stage we performed the hypergeometric test to assess if genes in this stage were significantly enriched in TFs and HCNEs.

## Figures and Tables

**Table 1:** Enriched biological process terms for stage-specific genes in preimplantation embryonic development. Similar function annotation clustering was presented by one typical function term. The last column stands for the validation of corresponding term in other studies of human preimplantation embryonic development.

Stage	Functional term	Bonferroni *P* value	Consistence with previous report
Oocyte	DNA repair	3.35*E* − 4	
	Cell cycle	2.92*E* − 3	[11, 12]
	DNA metabolic process	7.30*E* − 3	

Zygote	Cell cycle	3.33*E* − 4	[12]

2-cell	Posttranscriptional regulation of gene expression	3.56*E* − 2	

4-cell	Transcription	1.76*E* − 4	[11, 12]
	Regulation of transcription	6.34*E* − 3	[11, 12]
	Small GTPase mediated signal transduction	3.14*E* − 2	[11]

8-cell	DNA packaging	8.09*E* − 6	
	*RNA processing *	9.27*E* − 5	[11]
	Transcription	1.02*E* − 4	[11, 12]
	*Regulation of transcription *	3.08*E* − 4	[11, 12]
	*Translational elongation *		
	*Ribonucleoprotein complex biogenesis *	5.89*E* − 4	[12]
	*RNA elongation *	2.09*E* − 3	

Morulae	RNA processing	2.38*E* − 8	[11]
	Transcription	7.33*E* − 8	[11, 12]
	Regulation of transcription	5.38*E* − 7	[11, 12]
	*Ribonucleoprotein complex biogenesis *	9.81*E* − 6	[12]

Late blastocyst	Generation of precursor metabolites and energy	7.78*E* − 18	[12]
	Translation	4.11*E* − 12	[12]
	Oxidative phosphorylation	9.24*E* − 12	[12]
	Cellular respiration	1.69*E* − 8	[12]
	*Membrane organization *	9.28*E* − 6	
	*Protein localization *	2.74*E* − 5	
	Cofactor metabolic process	3.64*E* − 5	
	Protein transport	6.17*E* − 5	
	*Monosaccharide metabolic process *	3.67*E* − 3	
